# A Comparison of Muscle Activity Between the Cambered and Standard Bar During the Bench Press Exercise

**DOI:** 10.3389/fphys.2020.00875

**Published:** 2020-07-16

**Authors:** Michal Krzysztofik, Artur Golas, Michal Wilk, Petr Stastny, Robert George Lockie, Adam Zajac

**Affiliations:** ^1^Institute of Sport Sciences, The Jerzy Kukuczka Academy of Physical Education in Katowice, Katowice, Poland; ^2^Department of Sport Games, Faculty of Physical Education and Sport, Charles University, Prague, Czechia; ^3^Department of Kinesiology, California State University, Fullerton, Fullerton, CA, United States

**Keywords:** EMG, range of motion, internal movement structure, resistance training, training equipment

## Abstract

The aim of this study was to compare the electromyographic activity between the standard and cambered bar during the bench press (BP) exercise. Twelve resistance-trained males performed the flat BP with a standard and a cambered bar at selected loads (50%, 70%, and 90% 1RM). Muscle activation assessed by surface electromyography (sEMG) was recorded for the pectoralis major, anterior deltoid, and the lateral and long heads of the triceps brachii during each attempt. A three-way repeated measures ANOVA indicated statistically significant main interaction for bar × load × muscle (*p* < 0.01); bar × load (*p* < 0.01); bar × muscle (*p* < 0.01); load × muscle (*p* < 0.01). There was also a main effect for the bar (*p* < 0.01); load (*p* < 0.01); and muscle group (*p* < 0.01). The *post hoc* analysis for the main multiple interaction effect of bar × load × muscle showed a statistically significant increase in sEMG for the standard bar in the pectoralis major compared to the cambered bar at 50% 1RM (*p* < 0.01) and 90% 1RM (*p* < 0.01), as well as in the triceps brachii long at 90% 1RM (*p* < 0.01). Furthermore, a statistically significant decrease in sEMG was registered for the standard bar in the anterior deltoid compared to the cambered bar at 90% 1RM (*p* = 0.02). The results indicated that the cambered bar was superior in activating the anterior deltoid muscle compared to the standard bar during the BP exercise, whereas the standard bar provided higher pectoralis major and triceps brachii long head sEMG activity at 90% 1RM.

## Introduction

Resistance training is a common form of exercise for athletes and physically active people. A large and growing body of research has investigated the influence of the range of motion (ROM) during different resistance exercises and the specific neuromuscular adaptations they induce (Hartmann et al., 2012; Bloomquist et al., 2013; Martínez-Cava et al., 2019; Pallarés et al., 2020; Schoenfeld and Grgic, 2020). The available research has analyzed the effects of full (allowed by the standard barbell) or partial ROM during the bench press (BP) exercise on acute (Krzysztofik et al., 2020) as well as chronic changes in performance (Martínez-Cava et al., 2019). Moreover, the manipulation of the ROM during resistance exercises is a strategy commonly used among strength-trained athletes according to the principle of specificity (Schoenfeld and Grgic, 2020). However, recent evidence indicates that performing resistance exercises with a full ROM provides a greater stimulus, and leads to greater performance improvements in comparison to lifting with a limited ROM (Hartmann et al., 2012; Bloomquist et al., 2013; Martínez-Cava et al., 2019; Pallarés et al., 2020). Furthermore, Martínez-Cava et al. (2019) found that the greater the ROM used during the BP exercise, the greater improvements in maximum strength, and in the whole load-velocity spectrum in recreational and well-trained athletes.

Changes in the type or technique of exercise, ROM, external load, and movement tempo affect the physiological attributes of the muscles and acute fatigue experienced during resistance training. The above-mentioned factors most likely influence activation of muscles, which can be accessed via surface electromyography (sEMG) (Farina et al., 2002; Mills, 2005). Previous studies have examined the performance of particular BP variations (different grip widths and incline/decline bench positions) (Saeterbakken et al., 2017), comparison of successful and unsuccessful attempts (Tillaar and Ettema, 2009), sEMG activity of muscles changes surrounding the sticking point region (Tillaar and Ettema, 2010; Tillaar and Saeterbakken, 2012, 2013; Tillaar et al., 2012), and the influence of different loads and speeds of movement (Sakamoto and Sinclair, 2012; Van den Tillaar and Sousa, 2019). Evidence related to the influence of different ROM on muscle activity during the BP is limited to the analysis of particular parts of the concentric phase of the lift (Tillaar and Ettema, 2013). Since, the spatial relationship between the muscle origins and insertions change in accordance with joint motion, the muscle lines of action and moment arms must also change through the ROM (Krevolin et al., 2004). The muscle moment arm represents the mechanical advantage of a muscle and largely determines its role, for example, as a stabilizer or a prime mover (Ackland and Pandy, 2009). During the flat BP, the pectoralis major, anterior deltoid and triceps brachii are determined as the primary movers (Stastny et al., 2017). The study by Tillaar and Ettema (2013) indicated that sEMG activity of the pectoralis major was higher in the upper part of the concentric phase of the flat BP (in and post the sticking region) compared to the bottom part (pre to the sticking region) at 100% 1RM. Additionally, sEMG activity of the medial and lateral triceps heads was lower in the bottom part (pre to the sticking region) when compared to the upper one. Conversely, sEMG activity of the anterior deltoid was higher at the bottom part in comparison to the upper part of the lift. Similar results were reported by Krol and Golas (2017), who found decreased pectoralis major and the long head triceps with greater anterior deltoid brachii sEMG activity at the very beginning (from start to 25% of the lift) of the concentric phase of movement. However, this finding was observed only at 100% 1RM but not at submaximal loads (70, 80, 90% 1RM). Those findings may suggest that pectoralis major and triceps brachii are responsible for surpassing the sticking region and upper part, while anterior deltoid for initiating the bottom part of the BP.

According to Kandel et al. (2013) a higher load requires greater muscle activity to generate the required amount of force. The net force exerted by a muscle depends on the amount and rate coding of motor unit activity, the contractile properties of the activated muscle fibers, and the mechanical characteristics of the connective tissue that transmit muscle fiber forces to the skeleton (Enoka and Duchateau, 2015). Sakamoto and Sinclair (2012) showed that sEMG activity recorded from the pectoralis major, anterior deltoid, and triceps brachii was greater for heavier loads compared with lower loads, for the faster movement tempo compared to the slower one. Furthermore, the authors reported changes in sEMG of measured muscles between divided parts of the concentric phase of the BP. Similarly, the study by Krol and Golas (2017) indicated that the sEMG activity of the BP prime movers increased along with greater loads (from 70 to 100% 1RM). However, at maximal attempts, the role of the involved muscles can change in a particular phase of the movement (Krol and Golas, 2017). The pectoralis major muscle changes from a prime mover to a supportive prime-mover, and a greater involvement of the triceps brachii and anterior deltoid occurs at maximal effort (Krol and Golas, 2017). Results of the above-mentioned studies show the combined effects of the ROM and external load on sEMG activity of the prime movers during the BP exercise.

**FIGURE 1:**
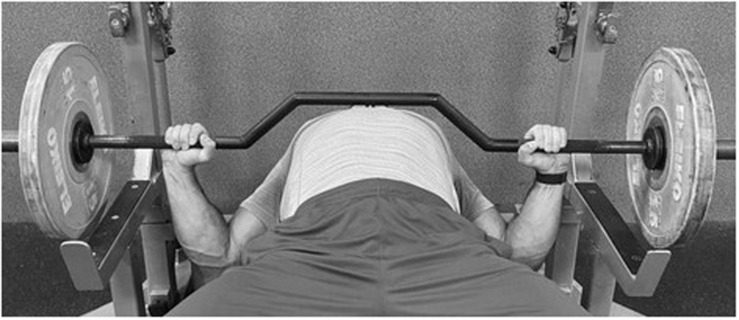
The cambered bar bench press.

Competitive athletes are increasingly using advanced resistance training techniques and methods to provide an additional stimulus to break through plateaus, prevent monotony, consolidate different training goals, or reduce the time of training sessions (Krzysztofik et al., 2019). The alternative or a supplementary tool which can be used during the BP includes the cambered bar. The cambered bar has a U-shape (camber; Figure 1) that can be used for the flat BP as it allows for a greater ROM by creating extra space for the torso allowing the hands to go deeper below the chest than with the standard bar (Corey, 1991), what may increase the use of elastic energy from the stretch and shortening cycle (SSC) (Turner and Jeffreys, 2010). Accordingly, the cambered bar BP features a greater overall increase in the ROM when compared with standard bar (Corey, 1991). The cambered bar allows to increase the ROM in an equal way to the dumbbells, however, requires less stabilization of the shoulder musculature than during the dumbbell BP, yet it demands similar flexibility from the athlete. Taking into consideration the results of the afore-mentioned studies, it could be suggested that the greater ROM during the flat BP reached by the use of the cambered bar could alter sEMG activity of the prime movers. However, there is no scientific research that has investigated the kinematics and kinetics during the cambered bar BP, and none has compared the changes in sEMG activity of the prime movers between the standard and cambered bar BP under different external loads.

Due the fact that all available EMG data is related to the BP exercise performed with the standard bar (Stastny et al., 2017), and there is no data related to sEMG activity of the prime movers during the BP performed with the cambered bar, the aim of this study was to compare the sEMG activity of muscles between the standard and cambered bar during the flat BP movement with different external loads. Since, the construction of the cambered bar increases the ROM during the BP and allows for a greater stretch of the muscles, a decrease in muscle activity of the prime movers may occur due to the fact, that part of the mechanical energy is stored in elastic components, such as tendons and ligaments. We hypothesized that sEMG activity of the prime movers, would be significantly greater in the flat BP performed with the standard bar compared to the cambered one.

## Materials and Methods

### Participants

Twelve healthy male subjects (age 26.9 ± 3.8 years, body mass 100.2 ± 10.5 kg) participated in the study. The average resistance training experience of the participants was 3.3 ± 1.3 years. Furthermore, the participants did not report any subjective evidence of musculoskeletal disorders and were able to perform the flat BP with a load greater than 120% body mass (123 ± 11.4 kg). All study participants were informed verbally and in writing about the procedures, possible risks and benefits of the tests, and provided written consent before the commencement of the experiment. The measurements were performed in the Strength and Power Laboratory of the Academy of Physical Education in Katowice. The study received the approval of the Bioethics Committee at the Academy of Physical Education in Katowice, Poland (10/2018), and was performed according to the ethical standards of the Declaration of World Medical Association, 2013.

### Study Design and Procedure

Four weeks before the experiment, the participants completed a familiarization session with the cambered bar BP to eliminate the learning effect which could influence their performance during the experimental session. Afterward, the participants attended two testing sessions, which were conducted at the same time of the day (in the morning between 9:00 am and 11:00 am) separated by a 1-week interval to avoid circadian variation. The first session was used to determine the one-repetition maximum (1RM) load of the flat BP with the standard bar. The second consisted of performing the BP exercise in a random order between the standard and cambered bar, with progressive loads (50%, 70%, and 90% 1RM) to record peak sEMG activity of the: anterior deltoid, pectoralis major, triceps brachii lateral head, triceps brachii long head. The participants did not perform any additional resistance exercises for 72 h prior to testing to avoid fatigue. Additionally, they were asked to maintain their dietary and sleep habits, refrain from alcohol consumption and not to take any medications or dietary supplements, as well as other ergogenic aids for 24 h prior to, and throughout the study.

### One-Repetition Maximum Strength Test

The 1RM test was conducted only for the standard bar. A standardized warm-up protocol was used for each session, including a general warm-up of approximately 5 min using a hand cycle ergometer (heart rate of around 130 bpm), followed by dynamic mobility exercises for the upper-body. During the specific warm-up, the participants performed 15 repetitions at 20% of their estimated 1RM followed by 10 repetitions at 40% 1RM, five repetitions at 60% 1RM, and three repetitions at 70% 1RM of the BP exercise with a 2/0/X/0 tempo of movement. The sequence of digits describing the tempo of movement (2/0/X/0) referred to a 2 s eccentric phase, 0 represented no pause during the transition phase, X referred to the maximum possible speed of movement during the concentric phase, and the last digit indicated no pause at the end of movement (Wilk et al., 2018). Next, the participants executed single repetitions of the BP exercise at their estimated 80% 1RM with a 5-min rest interval between successful trials. The load for each subsequent attempt was increased by 2.5–10 kg, and the process was repeated until failure. All 1RM values were obtained within five attempts. Hand placement on the barbell was individually selected which represented a grip width on the barbell of ∼150% individual bi-acromial distance. The positioning of the hands was recorded to ensure consistent hand placement during all testing sessions. No BP shirts, weightlifting belts or other supportive garments were permitted. All repetitions were performed without bouncing the barbell off the chest, without intentionally pausing at the transition between the eccentric and concentric phases, and without raising the hips off the bench. Two spotters were present during all attempts to ensure safety and technical proficiency.

### Experimental Session

The general warm-up for the experimental session was identical to the one used during the 1RM test. After warming-up, the participants performed one repetition of the BP exercise with the standard and cambered bar in a random order with progressively increasing loads (50%, 70%, and 90% 1RM) with a 3-min rest interval between successive attempts. The participants were asked to execute each repetition with full eccentric (lower the bar to the chest) and concentric phase (press it to full elbow extension) of the BP with the same tempo of movement as during the 1RM test (2/0/X/0) (Wilk et al., 2020a, b). For the purpose of the present study, a linear position transducer system (Tendo Power Analyzer, Tendo Sport Machines, Trencin, Slovakia) was used for the evaluation of differences in the ROM during the standard and cambered bar BP. The system consists of a displacement sensor connected to the bar by a kevlar cable which, through an interface, instantly transmits data to specific software (Tendo Power Analyzer Software 5.0).

### Electromyography

An eight-channel Noraxon TeleMyo 2400 system (Noraxon USA Inc., Scottsdale, AZ, United States; 1500 Hz) which was used for recording and analysis sEMG from the muscles during each repetition of the flat BP performed with a standard and cambered bar. The sEMG activity was recorded for four muscles: anterior deltoid, pectoralis major, triceps brachii lateral head, triceps brachii long head. Before placing the gel-coated self-adhesive electrodes (Dri-Stick Silver circular sEMG Electrodes AE-131, NeuroDyne Medical, United States), the skin was shaved, abraded, and washed with alcohol. The electrodes (11 mm contact diameter and a 2 cm center-to-center distance) were placed along the presumed direction of the underlying muscle fiber according to the recommendations by SENIAM (Hermens et al., 2000). The pectoralis major electrodes were placed on sterno-costal fibers, 4 cm medial to the axillary fold, the anterior deltoid electrodes were placed 1.5 cm distal and anterior to the acromion, and the triceps brachii electrodes were placed medial and inferior over the muscle belly for the long and lateral head. The EMG signals were sampled at a rate of 1000 Hz. Signals were band pass filtered with a cut off frequency of 8 Hz and 450 Hz, after which the root-mean-square (RMS) was calculated. The grounding electrode was placed on the medial part of the clavicle. Video recording was used (Logitech HD Pro C920 Pleasanton, United States) for identification of the beginning and completion of the movement. The maximal voluntary contraction (MVC) of each studied muscle was then recorded and selected to normalize the sEMG results. The normalization procedure was conducted for the dominant and non-dominant side of the body separately (to estimate peak maximum voluntary contraction values) and expressed as a percentage of MVC. To do so, two maximum isometric contractions were performed for 3 s with a 10 s rest interval between contractions, and 2 min between the MVC evaluation of each muscle according with SENIAM procedures (Hermens et al., 2000). Positions for the MVC were chosen in accordance with standardized procedures, based on commonly used testing positions for the prime movers of the BP exercise (Stastny et al., 2017). The triceps brachii lateral head, triceps brachii long head MVC test was obtained during the lying triceps extension with a 90° elbow flexion, the anterior deltoid MVC at 90° seated arm flexion, and the pectoralis major MVC during an isometric BP at 90° elbow flexion. All MVC tests were performed against a fixed multi-press bar. The analysis was based on peak sEMG activity of muscles during the BP movement from both the eccentric and concentric phases.

### Statistical Analysis

The data were processed using Statistica 9.1 software and presented as means ± standard deviations. All variables presented a normal distribution according to the Shapiro–Wilk test. The ROM differences between standard and cambered bar were assessed using paired *t*-tests. The effect of interactions between bar (standard vs. cambered), load (50% vs. 70% vs. 90% 1RM), and muscle activity (anterior deltoid vs. pectoralis major vs. triceps brachii lateral head vs. triceps brachii long head) were assessed using a three-way 2 × 3 × 4 (bar × load × muscle) repeated measures analysis of variance (ANOVA). In the event of a significant main effect, *post hoc* comparisons were conducted using the Tukey’s test. Statistical significance was set at *p* < 0.05. 95% confidence intervals and effect sizes were also calculated. Effect sizes (Cohen’s d) were reported where appropriate and interpreted as large (*d* ≥ 0.80); moderate (d between 0.79 and 0.50); small (d between 0.49 and 0.20) and trivial (*d* < 0.20) (Cohen, 2013).

## Results

The *t*-test data showed statistically significant differences in the ROM between the standard and cambered bar (380 ± 30 mm vs. 490 ± 20 mm, respectively; *p* < 0.01).

The three-way repeated measures ANOVA indicated statistically significant main interaction for bar × load × muscle group; bar × load; bar × muscle group; load × muscle group (*p* < 0.01 for all). The main differences were also observed for bar; load; and muscle group (*p* < 0.01 for all).

**FIGURE 2:**
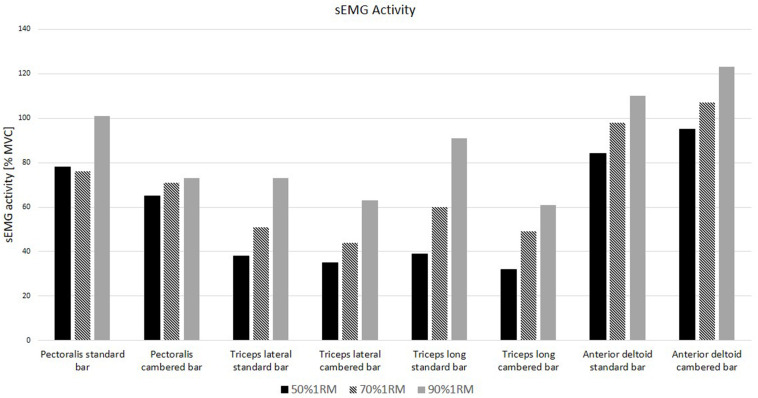
sEMG activity of selected muscles during bench press under different external loads.

The *post hoc* analysis for the main multiple interaction effect of bar × load × muscle group showed a statistically significant increase in sEMG activity for the standard bar in the pectoralis major compared to the cambered bar at a load of 50% 1RM (*p* < 0.01) and 90% 1RM (*p* < 0.01), as well as in the triceps brachii long head at 90% 1RM (*p* < 0.01). Furthermore, a statistically significant decrease in sEMG activity for the standard bar was observed in the anterior deltoid compared to the cambered bar at 90% 1RM (*p* = 0.02; Table 1 and Figure 2).

**TABLE 1:** The sEMG activity of muscles during the bench press exercises with standard and cambered bar and different external loads.

Muscle group	sEMG activity of muscles for standard bar [%MVC] (95% CI)	sEMG activity of muscles for cambered bar [%MVC] (95% CI)	*p*	ES
**50% 1RM**				
Anterior deltoid	84 ± 21	95 ± 13	0.06	0.63 moderate
	(70–98)	(87–104)		
Pectoralis major	78 ± 22	65 ± 18	0.01*	0.65 moderate
	(64–91)	(54–77)		
Triceps brachii lateral head	38 ± 6	35 ± 9	0.99	0.39 small
	(34–42)	(30–41)		
Triceps brachii long head	39 ± 15	32 ± 11	0.79	0.53 moderate
	(30–49)	(25–39)		
**70% 1RM**				
Anterior deltoid	98 ± 23	107 ± 18	0.19	0.44 small
	(83–112)	(96–119)		
Pectoralis major	76 ± 26	71 ± 21	0.99	0.21 small
	(60–93)	(58–85)		
Triceps brachii lateral head	51 ± 7	44 ± 9	0.72	0.87 large
	(47–56)	(38–50)		
Triceps brachii long head	60 ± 13	49 ± 17	0.09	0.73 moderate
	(52–68)	(38–60)		
**90% 1RM**				
Anterior deltoid	110 ± 12	123 ± 14	0.02*	0.90 large
	(102–118)	(114–131)		
Pectoralis major	101 ± 15	73 ± 22	0.01*	1.49 large
	(91–111)	(59–86)		
Triceps brachii lateral head	73 ± 9	63 ± 7	0.09	1.24 large
	(68–79)	(58–67)		
Triceps brachii long head	91 ± 9	61 ± 12	0.01*	2.83 large
	(86–97)	(53–69)		

Data presented as mean ± standard deviation; *statistically significant differences at p < 0.05; CI, Confidence interval.

The *post hoc* analysis for the main interaction effect of bar × muscle group showed a statistically significant increase in sEMG activity for the standard bar compared to the cambered bar in the pectoralis major (*p* < 0.01) and in the triceps brachii long head (*p* < 0.01). A significant decrease in sEMG activity for the standard bar compared to the cambered bar was registered in the anterior deltoid muscle (*p* < 0.01; Table 2).

**TABLE 2:** The result of main differences in sEMG activity of muscles during the bench press exercises at loads 50%, 70%, 90% 1RM with standard and cambered bar.

Muscle	sEMG activity of muscles for standard bar [%MVC]	sEMG activity of muscles for cambered bar [%MVC]	*p*	ES
Anterior deltoid	97 ± 22	108 ± 19	0.01*	0.54 moderate
Pectoralis major	85 ± 24	70 ± 20	0.01*	0.68 moderate
Triceps brachii lateral	54 ± 16	47 ± 14	0.28	0.48 small
Triceps brachii long	64 ± 25	47 ± 18	0.01*	0.78 moderate

Data presented as mean ± standard deviation; *statistically significant differences at p < 0.05.

The *post hoc* analysis for the main interaction effect of bar × load showed a statistically significant increase in sEMG activity for the standard bar compared to the cambered bar at a load of 90% 1RM (*p* < 0.01; Table 3).

**TABLE 3:** The result of main differences in sEMG activity of muscles between used loads.

Load	sEMG activity of 4 muscles for standard bar [%MVC]	sEMG activity of 4 muscles for cambered bar [%MVC]	*p*	ES
50% 1RM	60 ± 27	57 ± 29	0.61	0.11 trivial
70% 1RM	71 ± 25	68 ± 30	0.46	0.11 trivial
90% 1RM	94 ± 18	80 ± 29	0.01*	0.58 moderate

Data presented as mean ± standard deviation; *statistically significant differences at p < 0.05.

## Discussion

The BP exercise as one of the most popular resistance exercises for the upper body is most often performed with a standard bar. Since the path of the standard barbell limits the stretch of the involved muscles, resistance trained athletes often use the dumbbell BP exercise to increase the ROM in the shoulder joint (Cronin et al., 2001; Stastny et al., 2017). However, use of dumbbells instead of the barbell during the BP demands greater stabilization of the shoulder muscles (Saeterbakken et al., 2011). Considering that all available muscle sEMG data is related to the BP exercise performed with the standard bar, the aim of this study was to compare the sEMG activity of muscles between the standard and cambered bar during the flat BP exercise with different external loads.

The main finding of the study was a significant interaction between the effects of bars and loads on muscle sEMG activity of the prime movers. Thus, the obtained results indicated a significant difference in sEMG activity of all studied muscles between the cambered and standard bar during the BP exercise, which was dependent on the applied external load. The results of our study showed a significant increase in sEMG activity of the prime movers for the standard bar compared to the cambered bar, which confirmed our hypothesis. Despite the fact that the results of the presented study showed significantly higher sEMG activity for the four selected muscles during the standard bar BP, such changes were observed only at 90% 1RM. There were no significant differences in sEMG activity of the prime movers at 50% 1RM and 70% 1RM, what indicated that only high external loads differentiated sEMG activity of the prime movers between the standard and cambered bar. This confirms, that factors such as the load lifted and the ROM, affect muscle activity patterns during resistance exercises to maintain force output (Caterisano et al., 2002; Wilk et al., 2020c).

The greater ROM in the cambered bar likely caused greater storage and release of elastic energy from the pre-stretch mechanism, which may allow lifting a particular load during the BP exercise with lower sEMG activity of the prime movers (Turner and Jeffreys, 2010). Kawakami et al. (2002) and Padulo et al. (2013) showed reduced sEMG activity in SSC contractions compared to the solely concentric or eccentric contractions. This can partially explain the lower sEMG activity of prime movers during the cambered bar, as compared to the standard one. The increase in the ROM during the cambered bar is related with the bottom part of the movement and a more intense stretch, what could stimulate muscle spindles to a greater extent, causing a more effective use of the SSC (Turner and Jeffreys, 2010). Furthermore, when the cambered bar was used during the BP exercise, and thus was performed with a greater ROM, part of the mechanical energy could be transferred to passive structures such as tendons and ligaments, which caused decreases in sEMG activity, especially in the pectoralis major muscle. Moreover, it has been confirmed that the increase of mechanical work in the SSC is dependent on the stretch amplitude (Cronin et al., 2001). Therefore, there is a range of diminishing returns, whereby once the eccentric phase reaches a critical threshold, the subsequent concentric phase exhibits no further increase in force and may even result in a decrease of force output (Turner and Jeffreys, 2010). This could partly explain the decrease in sEMG activity of the prime movers during the BP performed with the cambered bar. Therefore, it may be concluded that the increased ROM, which potentially allowed for a greater muscle stretch in the eccentric phase, consequently decreased sEMG activity of the prime movers, especially the pectoralis major muscle. This is consistent with the results of Tillaar and Ettema (2013). They indicated that sEMG activity of the pectoralis major and medial as well as the triceps brachii lateral head was lower in the bottom phase (pre to the sticking region) when compared to the upper phase of the flat BP (after the sticking region).

However, the analysis of the changes in sEMG activity of particular muscles showed a significantly lower activity for the pectoralis major and triceps brachii long head muscles during the BP exercise with the cambered bar compared to the standard bar only at 90% 1RM. In contrast, the sEMG activity of the anterior deltoid was significantly higher for the cambered bar, which can be related to increased shoulder horizontal flexion demands because of its greater stretch. At the bottom part of the lift, the shoulders may internally rotate to achieve the needed ROM, which could elicit greater anterior deltoid sEMG activity. Due to the fact that these differences in sEMG activity of the muscles occurred only at 90% 1RM, we may suggest, that the greater the external load, the more anterior deltoid muscle recruitment is required in order to complete the lift. Since there is a lack of scientific data dedicated to muscle activity during the cambered bar BP, and the fact that dumbbells allow to increase the ROM in a similar way, we decided to contextualize the current results with studies comparing the dumbbell BP exercise with the barbell BP. Interestingly, Farias et al. (2017) indicated that the dumbbell BP elicited significantly greater pectoralis major sEMG activity with no differences in anterior deltoid sEMG activity in comparison to the barbell BP at 10RM. While, studies by Saeterbakken et al. (2011) and Welsch et al. (2005) demonstrated that the pectoralis major and anterior deltoid reached approximately the same peak sEMG activity during the barbell and dumbbell BP exercise at 1RM and 6RM, respectively. Furthermore, Krol and Golas (2017), reported a decreased pectoralis major activity with greater anterior deltoid and TB activity at the beginning of the concentric phase of the BP movement at 100% 1RM in comparison to submaximal loads (70, 80, 90% 1RM). Results of the above-mentioned studies show that with increasing external loads, muscle recruitment shifts to the anterior deltoid, while the pectoralis major changes to being a supportive prime mover. Moreover, in the present study, the participants performed at the same absolute load (50% 1RM, 70% 1RM, 90% 1RM), while in the exercise protocol by Welsch et al. (2005), Saeterbakken et al. (2011), and Farias et al. (2017), relative external loads for each lift were used, and the dumbbell loads represented 63%, 83%, and 86% of the barbell load, respectively. It can be assumed that if the absolute loads would be used, even higher anterior deltoid sEMG activity would occur.

Neuromuscular activity is not only dependent on the co-activation of primary movers, but also on the activity of their antagonist muscles as stabilizers (Stastny et al., 2017). The studies by Welsch et al. (2005), Saeterbakken et al. (2011), and Farias et al. (2017), indicated higher sEMG activity of the biceps brachii during the dumbbell BP in comparison to the barbell BP was observed. Krosshaug (2012) indicated that the dumbbell BP enhances sEMG activity of the biceps brachii due to increases in the internal torque requirements of the shoulder stabilizing muscles. Moreover, Dunnick et al. (2015) found that the sEMG activity of the latissimus dorsi muscle was significantly higher at greater loads during the BP exercise (60% 1RM vs. 80% 1RM). Despite the fact that we did not record the sEMG activity of antagonist muscles it is possible to speculate that heavy-loaded cambered bar BP increases sEMG activity of secondary muscles used in the BP due to increased ROM. Thus, on the basis of this fact, the cambered bar led to greater biceps brachii sEMG activity in comparison to the standard bar, what was manifested by lower triceps brachii activity.

Taking into consideration the obtained results, the use of the cambered bar during the BP exercise shifts the emphasis from the pectoralis major and triceps brachii activity to the anterior deltoid to complete the lift. This suggests that the cambered bar is a more efficient anterior deltoid developer than the standard bar during the BP exercise, which seems to be particularly significant for disciplines that use the anterior deltoid muscle during sport specific movements. This includes actions such as striking, throwing, punching and hitting, which are mandatory in baseball, tennis, volleyball, and numerous combat sports. Therefore, the results of the current study indicate that the use of the cambered bar may be an effective modification of the BP exercise to additionally overload and develop the anterior deltoid muscle.

There are certain study limitations that should be acknowledged. One methodological limitation of this study is that the evaluation of the external structure of the movement (i.e., forces and movement torques) was not investigated, nor were the kinematics considered of the two BP lifts and absolute, rather than relative loads were used. Moreover, activity of antagonist muscles was not considered. Future research should investigate the biomechanics of the cambered bar BP in different populations of athletes, both female and male. Additionally, even though the participants in this study were resistance-trained men, the sample size was relatively small. Considering the results of our study, especially the greater ROM, there is justification to record activity of antagonist or stabilizer muscles when the cambered bar is used in exercise protocols.

### Practical Implications

The cambered bar was more effective in activating the anterior deltoid muscle than the standard bar during the BP exercise, which seems particularly important for sport disciplines that engage the anterior deltoid muscle during specific upper-body movements such as striking, throwing, punching, and hitting. On the other hand, the standard bar more efficiently activates pectoralis major and triceps brachii long head in comparison with the cambered bar BP. Thus, the cambered bar may be an effective modification of the BP exercise to additionally overload and develop the anterior deltoid muscle, while standard bar BP may be beneficial for those seeking to maximize the involvement of the pectoralis major and triceps brachii long. However, athletes with limited shoulder mobility and/or past shoulder injuries should use the cambered bar with caution, progressing slowly toward improving the ROM in this exercise.

## Conclusion

The results of the present study indicate the combined effects of the ROM and external load on sEMG activity of the prime movers during the BP exercise. The increased ROM due to the use of cambered bar was superior in activating the anterior deltoid muscle compared to the standard bar during the BP exercise, whereas the standard bar provided higher pectoralis major and triceps brachii long head sEMG activity at 90% 1RM.

## Data Availability Statement

All datasets presented in this study are included in the article/supplementary material.

## Ethics Statement

The study received the approval of the Bioethics Committee at the Academy of Physical Education in Katowice, Poland (10/2018). The patients/participants provided their written informed consent to participate in this study.

## Author Contributions

AG and AZ contributed to the study concept and design. AG, MW, and MK contributed to the acquisition of data. MW and PS contributed to the analysis and interpretation of data. MW, AG, and MK contributed to the drafting of manuscript. MW, AZ, and RL contributed to the critical revision. All authors contributed to the article and approved the submitted version.

## Conflict of Interest

The authors declare that the research was conducted in the absence of any commercial or financial relationships that could be construed as a potential conflict of interest.

## References

[B1] Ackland D. C. Pandy M. G. (2009). Lines of action and stabilizing potential of the shoulder musculature. *J. Anat.* 215 184–197. 10.1111/j.1469-7580.2009.01090.x 19490400 PMC2740966

[B2] Bloomquist K. Langberg H. Karlsen S. Madsgaard S. Boesen M. Raastad T. (2013). Effect of range of motion in heavy load squatting on muscle and tendon adaptations. *Eur. J. Appl. Physiol.* 113 2133–2142. 10.1007/s00421-013-2642-7 23604798

[B3] Caterisano A. Moss R. F. Pellinger T. K. Woodruff K. Lewis V. C. Booth W. (2002). The effect of back squat depth on the EMG activity of 4 superficial hip and thigh muscles. *J. Strength Cond. Res.* 16 428–432. 10.1519/00124278-200208000-00014 12173958

[B4] Cohen J. (2013). *Statistical Power Analysis for the Behavioral Sciences.* Burlington, NJ: Elsevier Science.

[B5] Corey S. W. (1991). The cambered bar. *Strength Cond. J.* 13 36–38.

[B6] Cronin J. B. McNair P. J. Marshall R. N. (2001). Magnitude and decay of stretch-induced enhancement of power output. *Eur. J. Appl. Physiol.* 84 575–581. 10.1007/s004210100433 11482554

[B7] Dunnick D. D. Brown L. E. Coburn J. W. Lynn S. K. Barillas S. R. (2015). Bench press upper-body muscle activation between stable and unstable loads. *J. Strength Cond. Res.* 29 3279–3283. 10.1519/JSC.0000000000001198 26540024

[B8] Enoka R. M. Duchateau J. (2015). Inappropriate interpretation of surface EMG signals and muscle fiber characteristics impedes understanding of the control of neuromuscular function. *J. Appl. Physiol.* 119 1516–1518. 10.1152/japplphysiol.00280.2015 26159758

[B9] Farias D. de A. Willardson J. M. Paz G. A. Bezerra E. de S. (2017). Maximal strength performance and muscle activation for the bench press and triceps extension exercises adopting dumbbell, barbell, and machine modalities over multiple sets. *J. Strength Cond. Res.* 31 1879–1887. 10.1519/JSC.0000000000001651 27669189

[B10] Farina D. Fosci M. Merletti R. (2002). Motor unit recruitment strategies investigated by surface EMG variables. *J. Appl. Physiol.* 92 235–247. 10.1152/jappl.2002.92.1.235 11744666

[B11] Hartmann H. Wirth K. Klusemann M. Dalic J. Matuschek C. Schmidtbleicher D. (2012). Influence of squatting depth on jumping performance. *J. Strength Cond. Res.* 26 3243–3261. 10.1519/JSC.0b013e31824ede62 22344055

[B12] Hermens H. J. Freriks B. Disselhorst-Klug C. Rau G. (2000). Development of recommendations for SEMG sensors and sensor placement procedures. *J. Electromyogr. Kinesiol.* 10 361–374. 10.1016/S1050-6411(00)00027-4 11018445

[B13] Kandel E. R. Jessell T. M. Schwartz J. H. Siegelbaum S. A. Hudspeth A. J. Mack S. (2013). *Principles of Neural Science*, 5th Edn New York, NY: McGraw-Hill.

[B14] Kawakami Y. Muraoka T. Ito S. Kanehisa H. Fukunaga T. (2002). In vivo muscle fibre behaviour during counter-movement exercise in humans reveals a significant role for tendon elasticity. *J. Physiol.* 540(Pt 2) 635–646. 10.1113/jphysiol.2001.013459 11956349 PMC2290252

[B15] Krevolin J. L. Pandy M. G. Pearce J. C. (2004). Moment arm of the patellar tendon in the human knee. *J. Biomech.* 37 785–788. 10.1016/j.jbiomech.2003.09.010 15047009

[B16] Krol H. Golas A. (2017). Effect of barbell weight on the structure of the flat bench press. *J. Strength Cond. Res.* 31 1321–1337. 10.1519/JSC.0000000000001816 28415066 PMC5400411

[B17] Krosshaug T. (2012). “Revealing “secrets” of strength training exercises with kinetic analyses,” in *Proceedings of the 8th International Conference on Strength Training (ICTS 2012)*, Oslo, 81–83.

[B18] Krzysztofik M. Wilk M. Golas A. Lockie R. G. Maszczyk A. Zajac A. (2020). Does eccentric-only and concentric-only activation increase power output? *Med. Sci. Sports Exerc.* 52 484–489. 10.1249/MSS.0000000000002131 31425385

[B19] Krzysztofik M. Wilk M. Wojdala G. Golas A. (2019). Maximizing muscle hypertrophy: a systematic review of advanced resistance training techniques and methods. *Int. J. Environ. Res. Public Health* 16:4897. 10.3390/ijerph16244897 31817252 PMC6950543

[B20] Martínez-Cava A. Hernández-Belmonte A. Courel-Ibáñez J. Morán-Navarro R. González-Badillo J. J. Pallarés J. G. (2019). Bench press at full range of motion produces greater neuromuscular adaptations than partial executions after prolonged resistance training. *J. Strength Cond. Res.* 10.1519/JSC.0000000000003391 [Epub ahead of print]. 31567719

[B21] Mills K. R. (2005). The basics of electromyography. *J. Neurol. Neurosurg. Psychiatry* 76 Suppl. 2(Suppl. 2) ii32–ii35. 10.1136/jnnp.2005.069211 15961866 PMC1765694

[B22] Padulo J. Tiloca A. Powell D. Granatelli G. Bianco A. Paoli A. (2013). EMG amplitude of the biceps femoris during jumping compared to landing movements. *SpringerPlus* 2:520. 10.1186/2193-1801-2-520 24156093 PMC3797910

[B23] Pallarés J. G. Cava A. M. Courel-Ibáñez J. González-Badillo J. J. Morán-Navarro R. (2020). Full squat produces greater neuromuscular and functional adaptations and lower pain than partial squats after prolonged resistance training. *Eur. J. Sport Sci.* 20 115–124. 10.1080/17461391.2019.1612952 31092132

[B24] Saeterbakken A. H. Mo D.-A. Scott S. Andersen V. (2017). The effects of bench press variations in competitive athletes on muscle activity and performance. *J. Hum. Kinet.* 57 61–71. 10.1515/hukin-2017-0047 28713459 PMC5504579

[B25] Saeterbakken A. H. Tillaar R. V. D. Fimland M. S. (2011). A comparison of muscle activity and 1-RM strength of three chest-press exercises with different stability requirements. *J. Sports Sci.* 29 533–538. 10.1080/02640414.2010.543916 21225489

[B26] Sakamoto A. Sinclair P. J. (2012). Muscle activations under varying lifting speeds and intensities during bench press. *Eur. J. Appl. Physiol.* 112 1015–1025. 10.1007/s00421-011-2059-0 21735215

[B27] Schoenfeld B. J. Grgic J. (2020). Effects of range of motion on muscle development during resistance training interventions: a systematic review. *SAGE Open Med.* 8:205031212090155. 10.1177/2050312120901559 32030125 PMC6977096

[B28] Stastny P. Golas A. Blazek D. Maszczyk A. Wilk M. Pietraszewski P. (2017). A systematic review of surface electromyography analyses of the bench press movement task. *PLoS One* 12:e0171632. 10.1371/journal.pone.0171632 28170449 PMC5295722

[B29] Tillaar R. Sæterbakken A. Ettema G. (2012). Is the sticking region in bench press the result of diminishing potentiation? *J. Sports Sci.* 30 591–599. 10.1080/02640414.2012.658844 22304656

[B30] Tillaar R. V. D. Ettema G. (2009). A comparison of kinematics and muscle activity between successful and unsuccessful attempts in bench press. *Med. Sci. Sports Exerc.* 41 2056–2063. 10.1249/MSS.0b013e3181a8c360 19812510

[B31] Tillaar R. V. D. Ettema G. (2010). The “sticking period” in bench press. *J. Sports Sci.* 28 529–535. 10.1080/02640411003628022 20373201

[B32] Tillaar R. V. D. Ettema G. (2013). A comparison of muscle activity in concentric and counter movement maximum bench press. *J. Hum. Kinet.* 38 63–71. 10.2478/hukin-2013-0046 24235985 PMC3827767

[B33] Tillaar R. V. D. Saeterbakken A. H. (2012). The sticking region in three chest-press exercises with increasing degrees of freedom. *J. Strength Cond. Res.* 26 2962–2969. 10.1519/JSC.0b013e3182443430 22158100

[B34] Tillaar R. V. D. Saeterbakken A. H. (2013). Fatigue effects upon sticking region and electromyography in a six-repetition maximum bench press. *J. Sports Sci.* 31 1823–1830. 10.1080/02640414.2013.803593 23879709

[B35] Turner A. N. Jeffreys I. (2010). The stretch-shortening cycle: proposed mechanisms and methods for enhancement. *Strength Cond. J.* 32 87–99. 10.1519/SSC.0b013e3181e928f9

[B36] Van den Tillaar R. Sousa C. (2019). Comparison of muscle activation and barbell kinematics during bench press with different loads. *AKUT* 25, 37–50. 10.12697/akut.2019.25.03

[B37] Welsch E. A. Bird M. Mayhew J. L. (2005). Electromyographic activity of the pectoralis major and anterior deltoid muscles during three upper-body lifts. *J. Strength Cond. Res.* 19 449–452. 10.1519/14513.1 15903389

[B38] Wilk M. Gepfert M. Krzysztofik M. Mostowik A. Filip A. Hajduk G. (2020a). Impact of duration of eccentric movement in the one-repetition maximum test result in the bench press among women. *J. Sports Sci. Med.* 19 317–322. 32390725 PMC7196738

[B39] Wilk M. Golas A. Stastny P. Nawrocka M. Krzysztofik M. Zajac A. (2018). Does tempo of resistance exercise impact training volume? *J. Hum. Kinet.* 62 241–250. 10.2478/hukin-2018-0034 29922395 PMC6006544

[B40] Wilk M. Golas A. Zmijewski P. Krzysztofik M. Filip A. Del Coso J. (2020b). the effects of the movement tempo on the one-repetition maximum bench press results. *J. Hum. Kinet.* 72 151–159. 10.2478/hukin-2020-0001 32269656 PMC7126254

[B41] Wilk M. Tufano J. J. Zajac A. (2020c). The influence of movement tempo on acute neuromuscular, hormonal, and mechanical responses to resistance exercise – a mini-review. *J. Strength Cond. Res.* 10.1519/JSC.0000000000003636 [Epub ahead of print]. 32735429

[B42] World Medical Association (2013). World medical association declaration of helsinki: ethical principles for medical research involving human subjects. *JAMA* 310:2191. 10.1001/jama.2013.281053 24141714

